# Student directed learning in medical neuroscience curricula

**DOI:** 10.5116/ijme.591b.1f43

**Published:** 2017-05-25

**Authors:** Douglas J. Gould, Misa Mi, Gustavo A. Patino

**Affiliations:** 1 Department of Biomedical Sciences, Oakland University William Beaumont School of Medicine, USA

**Keywords:** Student directed learning, medical neuroscience, curricula, USA

## Introduction

Student-directed learning (SDL) holds the promise of endowing medical students with the skills to tackle the growing body of information in medicine and facilitate its integration to solve clinical problems. The concept of SDL is not new, it grew out of a more commonly used and closely related concept: self-directed learning. Barrows characterizes SDL as the development of effective self-directed learning skills.^1^ Skills of self-assessment and SDL allow the student to become sensitive to personal learning needs and to locate and to use appropriate information resources. These are essential skills for doctors, as medical knowledge moves ever onwards and new problems will have to be understood in the care of patients.

The sufficiency of SDL experiences is one of the criteria used in the United States of America (US) by the Liaison Committee on Medical Education for accreditation (LCME). The LCME is the US Department of Education-recognized accrediting body for programs leading to the MD degree in the US One of its directives, ED-5-A, states that ‘self-assessment on learning needs; the independent identification, analysis and synthesis of relevant information; and the appraisal of the credibility of information sources’ is a necessary part of a medical education program.^2^ This is a lofty goal – allowing students to determine what content is necessary, identifying and learning said content and then conducting some-level of self-appraisal or assessment. Historically, those tasks are a primary function of the medical school itself, accomplished through its well-worn system of using external and internal indicators for what student’s learning needs are, the faculty then synthesizes and vets content, which is then presented to the student in a variety of ways (e.g. lecture), followed by an assessment which is also a traditional role of the faculty.

The origins of SDL are rooted in active learning (AL) where students engage in activities to promote higher-levels of learning and critical thinking. SDL values the progression up the Bloom’s taxonomic ladder as seen in AL, but with an increasing level of responsibility by the student to determine what and how to get there.^3^ It is in this light that SDL becomes not only a desired process, but an essential next step on the academic timeline. Traditionally, neuroscience is a subject perceived by physicians and medical students as very important and highly complex; as such, it could greatly benefit from SDL. The implementation of SDL has been hindered by the lack of standardized definitions and methods, lack of incentive to innovate and an overall persistent adherence to traditional methods. A first step in implementing SDL in medical neuroscience is to determine the amount and type of SDL that currently exists in neuroscience courses.

### SDL in Neuroscience Courses

We were able to find very few examples of SDL in medical school neuroscience courses described in the literature. In all cases reviewed, seven in total, course objectives were determined by the faculty with occasional input from the school administration, but never from the students. The timeline of the course was also determined in advance by the faculty and administration. In most cases, the materials were selected by the faculty as either required or recommended resources. Both formative and summative evaluations were reported. Four studies offered a description of their summative assessment. Formats included a mix of open ended questions and multiple choice questions, and written and lab examinations.^4^^-^^7^ No mention was made in any case of student participation in examination development.

Even though a consensus definition of SDL exists in the literature, the one provided by the LCME emphasizes the ability of students to identify their own learning objectives and source materials.^2^ Others expand the definition to include self-determination of timeline of the course and assessments, and type of assessment methods; along with self-monitoring activities, measurements of motivation and self-direction, and progressive participation of the students in the conduct of the course.^8^^,^^9^ Our study sought to identify and categorize what aspects of SDL have been implemented in neuroscience courses – in sum, the literature indicates that neuroscience programs include very limited SDL.

In all programs reviewed, the faculty and sometimes administrative staff, were in charge of determining the objectives and timeline of the course, session format and educational materials, and type and timing of the assessments. The only exceptions were PBL sessions, in which the students had to determine by themselves what they needed to learn and what sources of information they would use. Given the generally accepted content that medical students must master to demonstrate readiness for practice, as exemplified by the USMLE Step exams,^10^ it is unlikely that course objectives could be completely left up to students. As all the programs reviewed include lectures, the objectives of individual academic sessions are only partially set by students at best – in every instance.

In light of the complexity and substantial amount of content in neuroscience that medical students need to learn, there is a concern with the devolution by medical educators of responsibility to students for learning important neuroscience content on their own terms. Furthermore, undergraduate medical education must take place within a set amount of time, 4 years for most programs is the goal, with mounting pressure to reduce that time. This also severely limits the ability of transferring determination of the course timeline to the students.

### Implications

Health professionals are expected to be lifelong learners, faced with continuous expansion of the knowledge base. Self-direction for lifelong learning is becoming of paramount importance for health professionals. This study reveals the inadequacy of course design incorporating self-directed learning activities/strategies or adult learning principles in teaching medical students the content of neuroscience in preclinical medical education. Such inadequacy might be a consequence of the logistical limitations imposed on medical schools, as discussed previously. Nevertheless, for SDL to benefit students from their first exposure to the field of medical neuroscience it becomes important to identify strategies to incorporate SDL within those confines.

## Conclusions

Evidence on how SDL enhances students’ outcome-based learning and lifelong learning skills is still lacking. This study indicates scant research on how students organize, manage, do and judge their self-directed learning activities in the neuroscience curriculum. While the multiple characteristics of SDL described in the literature take full advantage of adult learner characteristics, their implementation within a structured and regulated environment like undergraduate medical education is limited. New educational pathway formats allow the incorporation of more elements of SDL, and the evolution of such paradigms might hold the key to eventually implement preclinical courses that are more fully SDL-oriented. 

### Conflict of Interest

The authors declare that they have no conflict of interest.
